# Penetrating Chest Trauma in a Child Caused by a Fall on a Metallic Bar: A Case Report

**DOI:** 10.7759/cureus.53729

**Published:** 2024-02-06

**Authors:** Hatim Jabri, Othmane Alaoui, Abdelhalim Mahmoudi, Khalid Khatalla, Youssef Bouabdallah

**Affiliations:** 1 Pediatric Surgery, Hassan II Hospital of Fez, Sidi Mohamed Ben Abdellah University, Faculty of Medicine and Pharmacy of Fez, Fez, MAR

**Keywords:** case report, ct chest, child, metallic bar, penetrating chest trauma

## Abstract

Penetrating chest trauma in children is an uncommon condition. Patients may be asymptomatic or in a critical state. Visceral and vascular damage are frequently present when penetrating objects enter the thoracic cavity. Although many studies have discussed penetrating thoracic trauma in adults, very few deal with the pediatric population.

Here, we present the case of a 13-year-old child with an intrathoracic metallic bar after penetrating chest trauma. The clinical examination showed a stable patient with a palpable bar and subcutaneous emphysema in the left axillary area. The radiological scan did not reveal any vital damage. The bar was removed through the entry wound without thoracotomy or thoracoscopy. The patient evolved without any incident and was discharged after three days. Good improvement was noted over three months of follow-up.

Intrathoracic foreign bodies secondary to penetrating trauma are rare in children. An exhaustive imaging examination is required to identify the precise location of the foreign material and find any severe organ or vascular injuries. If the condition permits, direct removal should be attempted in an operating room, in case surgical intervention is needed after the extraction.

## Introduction

Penetrating chest trauma is uncommon [1]. Its incidence remains unknown, with studies on this subject in children being rare. This case report presents an unusual case of penetrating thoracic trauma in a child.

Penetrating injuries can be mild or life-threatening, depending on the site, course of the penetrating object, and depth of the injury [2]. These lesions have a very high mortality rate due to the critical anatomical structures in the chest [2,3]. Efficient evaluation and identification of patients requiring surgical intervention is imperative. The approach in such circumstances must always consider the dangers associated with removing the object and the potential complications [4].

Here, we present an unusual case of a 13-year-old child who presented with penetrating chest trauma after falling on a metallic bar.

## Case presentation

**Figure 1:**
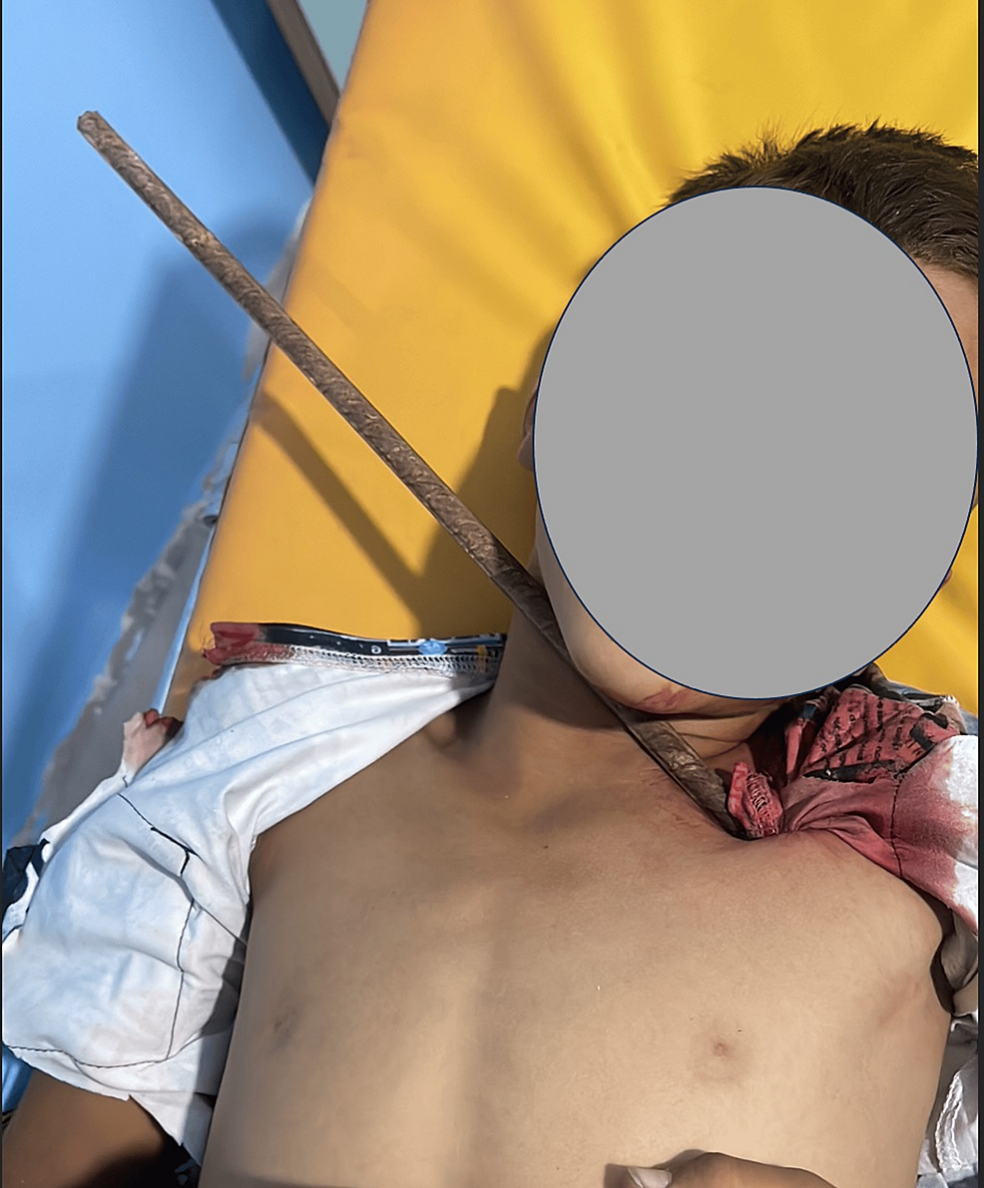
Clinical image showing the foreign body penetrating the chest.

A 13-year-old male presented to the emergency department with penetrating thoracic trauma caused by a metal bar after an accidental fall from a height of 2 m, with an entry from the left subclavian region without leaving the axillary region (Figure 1).

**Figure 2:**
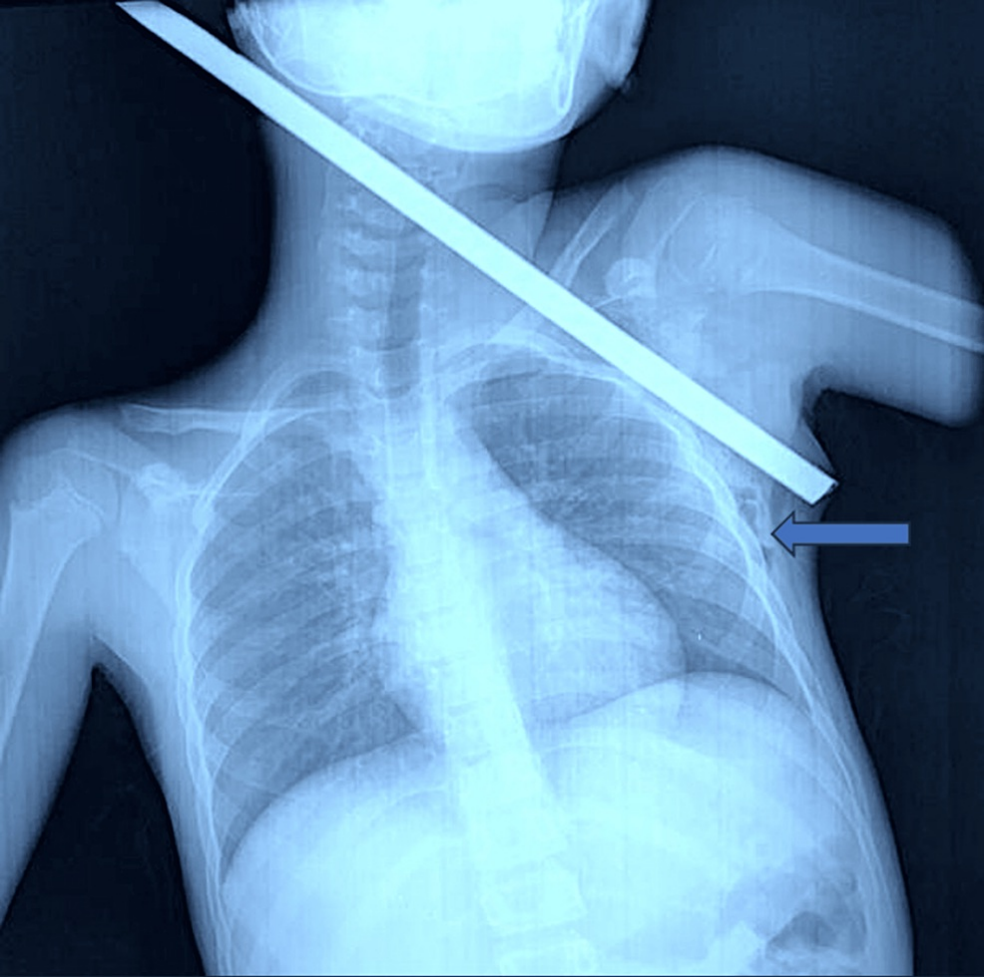
Chest X-ray showing a metallic rod penetrating through the left thorax. The blue arrow shows the subcutaneous emphysema.

Clinical examination identified a conscious patient. The patient had a respiratory rate of 20 breaths per minute, an oxyhemoglobin saturation of 98% in room air, a pulse of 98 beats per minute, and a blood pressure of 96/64 mmHg. There were no signs of active bleeding. A complete clinical examination revealed a palpable foreign body with subcutaneous emphysema in the left axillary region. The vascular and neurological examination of the left upper limb was normal, without other abnormalities. After conditioning and giving 500 mg of amoxicillin-clavulanic acid with an intramuscular injection of 1,500 IU of anti-tetanus serum, the patient underwent a chest X-ray, which showed the metal bar penetrating through the left thorax, without signs of pneumothorax or hemothorax (Figure 2).

**Figure 3:**
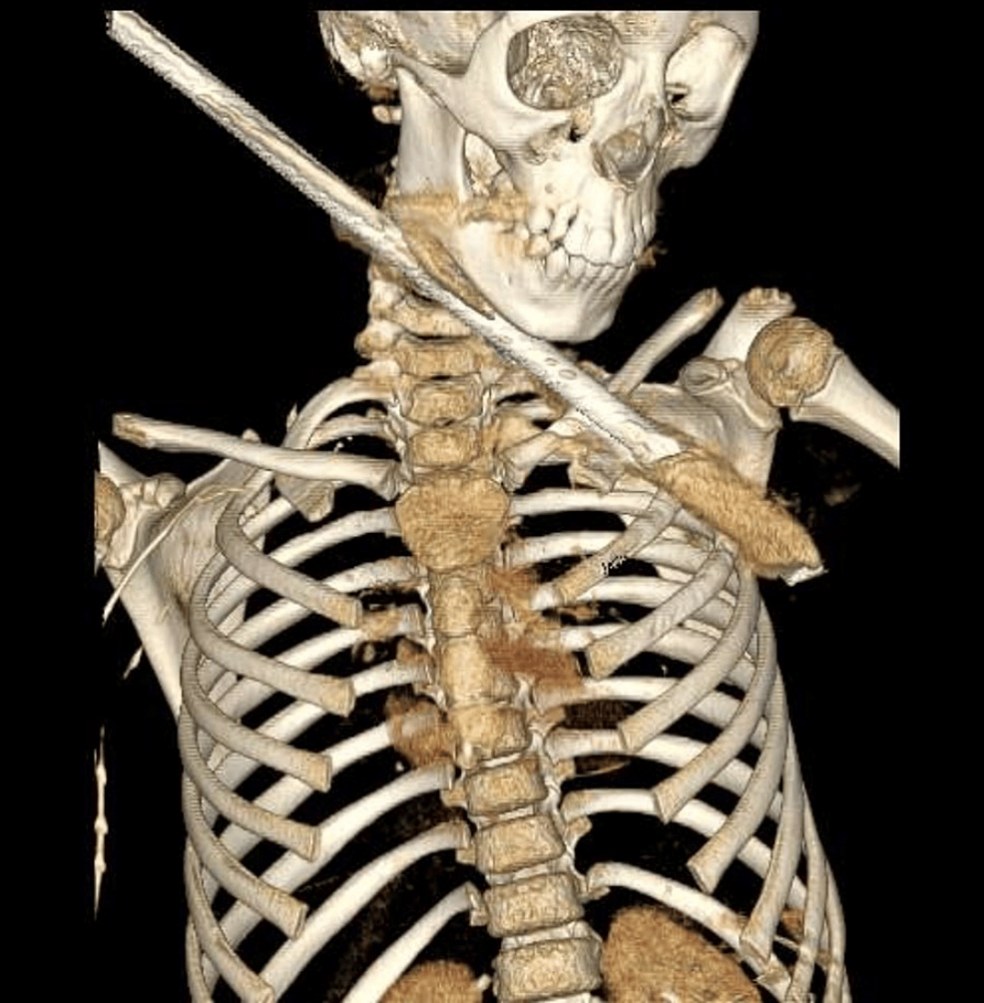
Three-dimensional visualization of the CT scan revealing a rod penetrating the left hemithorax through the middle part of the superior surface of the left clavicle and arriving at the axillary region.

We performed a CT angiography of the chest, which excluded pleural, pulmonary, cardiovascular, and neurovascular injuries evident in the three-dimensional reconstruction (Figure 3).

**Figure 4:**
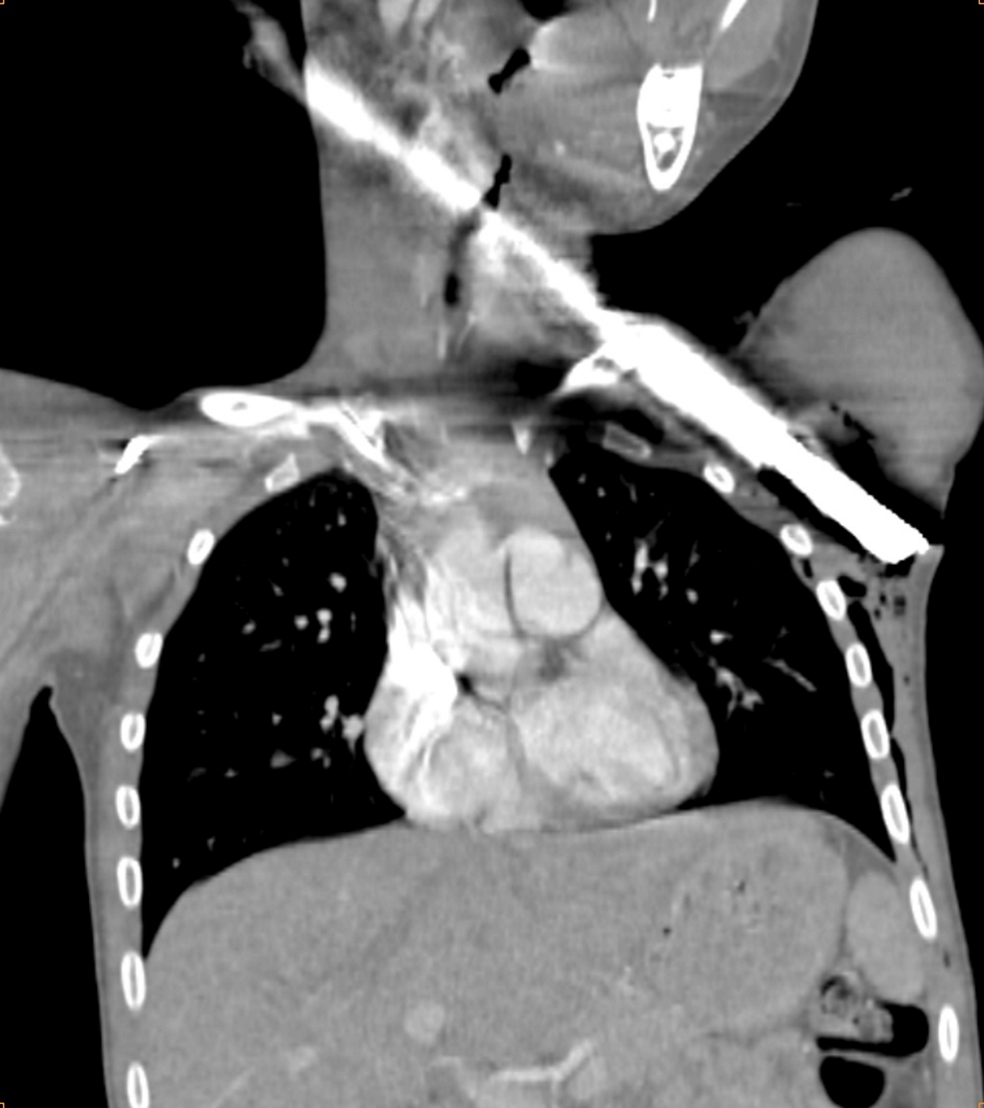
CT angiography of the chest showing the presence of subcutaneous emphysema in the soft tissues of the left posterolateral chest wall without major injuries.

However, we noted the presence of subcutaneous emphysema in the soft tissues of the left anterior and posterolateral chest wall (Figure 4).

**Figure 5:**
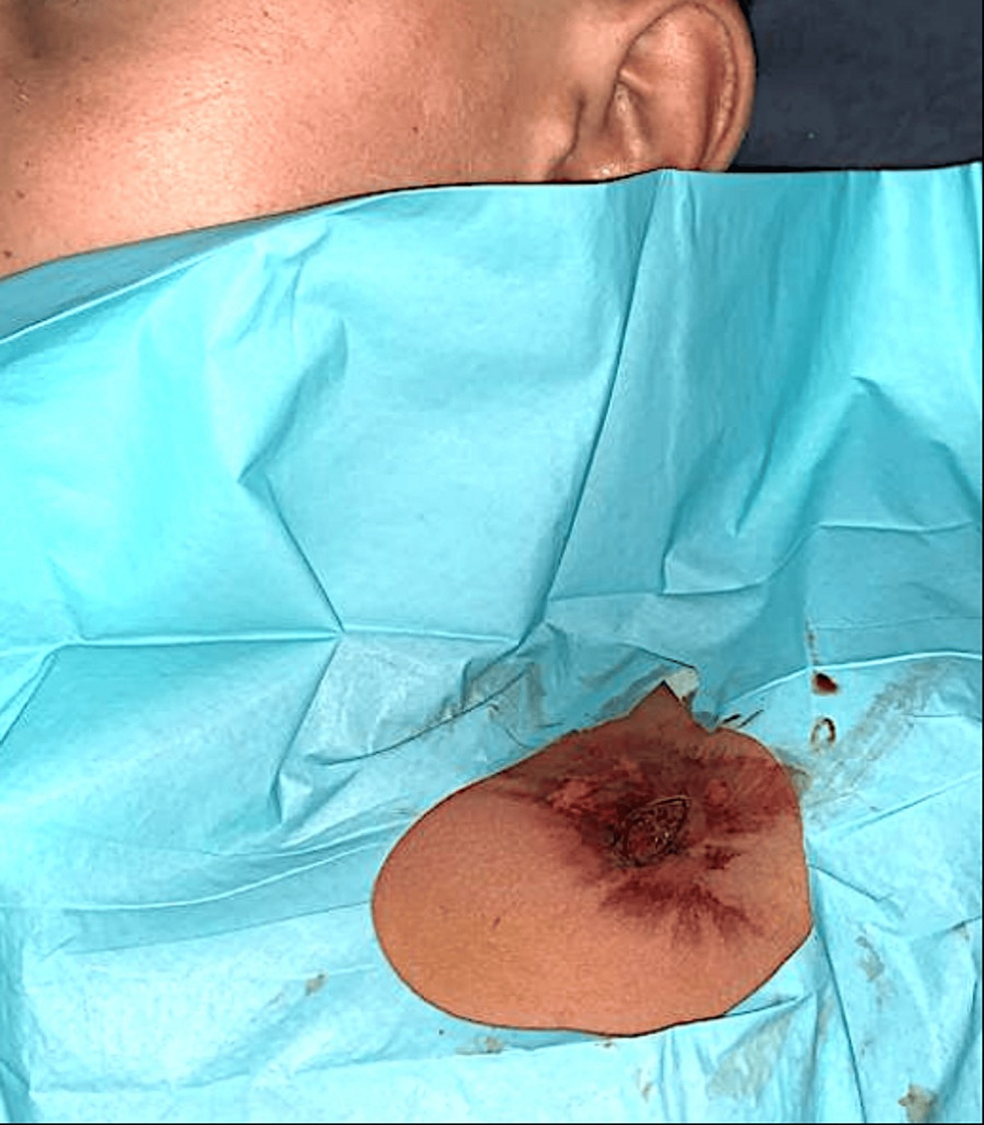
Post-extraction picture showing necrotic and missing skin surrounding the areas of entry.

Blood storage was done. Then, we elected to extract the rod through the entry site from the same tract as its entry and exit under local anesthesia by percutaneous injection of 4 mL of lidocaine 2% solution in the operating room, which was performed without incident. The vital parameters did not change, and emphysema remained constant. There was no visible bleeding; the pectoralis major was torn, with necrotic and missing skin surrounding the areas of entry (Figure 5).

**Figure 6:**
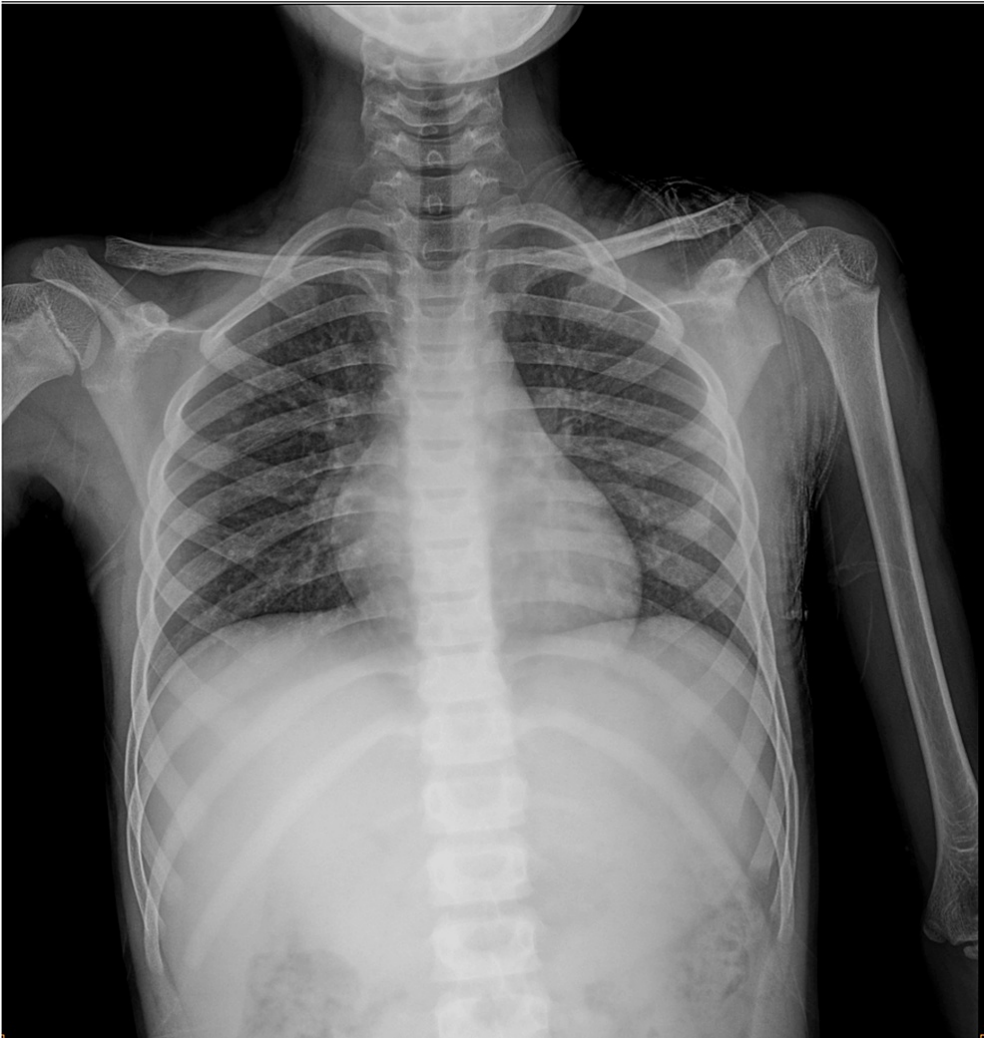
Post-extraction chest X-ray without abnormalities.

Betadine-soaked gauze was put into the open thoracic wound to avoid infection. The pectoralis major was sutured, the dead tissue was debrided, and a pressure dressing was applied. The patient was shifted immediately to the observation room. He underwent a chest X-ray two hours after extraction, and no pneumothorax was noted (Figure 6).

The patient was discharged from the hospital on the third day after a normal chest X-ray. After 10 days, no wound infection was noted. Regular clinical and radiological findings over three months showed good recovery.

## Discussion

Penetrating chest trauma represents a challenging situation. In children, this condition is rarely reported [5]. The guidelines for the management of penetrating chest trauma are as follows: avoid exploring the wound site, avoid removing the object without a complete check-up, and do so only when anesthesia and surgical support are available [2]. The treatment of thoracic wall foreign bodies remains controversial [1,6]. The thoracic angiogram provides a detailed analysis of the characteristics of the foreign body, its location, and associated organ damage. Fewer than 20% of patients require surgical treatment [7,8].

Thoracotomy has long been considered the standard treatment for major thoracic trauma involving foreign bodies [4]. However, the large incisions involved in thoracotomy might be associated with significant morbidity and hospitalization [4,9]. Since its appearance, thoracoscopy has become a safer approach for the management of stable patients, and thoracotomy is recommended in cases of hemodynamic instability [4].

The prognosis after penetrating thoracic trauma depends on the mechanism, location, associated injuries, hemodynamic status, appropriate imaging, and treatment [7].

According to Chen et al. [10], if it is confirmed that the foreign body has not caused organ, vessel, or nerve damage, surgical removal under local anesthesia should be considered; otherwise, general anesthesia and close hemodynamic monitoring should be considered.

Our patient had no vascular or organ damage. The first reason was that the metal bar was at a fortunate angle to the patient’s body. The second was that the cotton clothes around the bar provided soft protection for vessels. Therefore, after conditioning and performing a CT scan, which ruled out any severe lesions, the metallic bar was removed by just drawing it out. A postoperative chest X-ray confirmed the absence of complications.

Despite the particularity of this case, the principal limitation was the absence of vital damage, which made management less challenging.

## Conclusions

Penetrating thoracic injuries by metallic bars in children are uncommon. An initial evaluation and transfer to an appropriate center are necessary. In stable patients, it is necessary to complete an assessment of the trauma injuries to determine the position of the foreign material and identify potential complications. Then, if the condition permits, direct removal of the foreign body is possible.
